# Development and validation of an eco-friendly HPLC–UV method for determination of atorvastatin and vitamin D_3_ in pure form and pharmaceutical formulation

**DOI:** 10.1186/s13065-023-00975-6

**Published:** 2023-06-20

**Authors:** Khaled Maged, Magda M. El-Henawee, Soad S. Abd El-Hay

**Affiliations:** 1 Pharmaceutical Analytical Chemistry Department, Faculty of Pharmacy, Sinai University, El-Areesh, Egypt; 2 Analytical Chemistry Department, Faculty of Pharmacy, Zagazig University, Zagazig, 44519 Egypt

**Keywords:** Atorvastatin calcium, Vitamin D_3_, Pharmaceuticals, Green HPLC, Greenness assessment tools, GAPI, AGREE

## Abstract

Statin-associated muscle symptoms are considered as obvious adverse effects of prolonged statin therapy such as myopathy, myalgia, and rhabdomyolysis. These side effects are associated with vitamin D_3_ deficiency and can be adjusted by amendment of serum vitamin D_3_ level. Green chemistry aims to decrease the harmful effects of analytical procedures. Here we have developed a green and eco-friendly HPLC method for the determination of atorvastatin calcium and vitamin D_3_. The two drugs were separated in less than 10 min on Symmetry column C_18_ (100 × 4.6 mm, 3.5 µm) using a mixture consisting of 0.1% ortho-phosphoric acid (OPA) (pH = 2.16) and ethanol as the mobile phase in gradient manner. We have used Green Analytical Procedure Index (GAPI) tools and the Analytical GREEnness Metric Approach (AGREE) for assessment of the greenness of our proposed method. The method proved linearity over concentration ranges of (5–40) and (1–8) µg/ml with low limit of detection of 0.475 and 0.041 µg/ml for atorvastatin calcium and vitamin D_3_ respectively. The method was successfully validated in accordance with ICH instructions and utilized for determination of the drugs of interest either in pure form or in their pharmaceuticals.

## Introduction

**Fig. 1:**
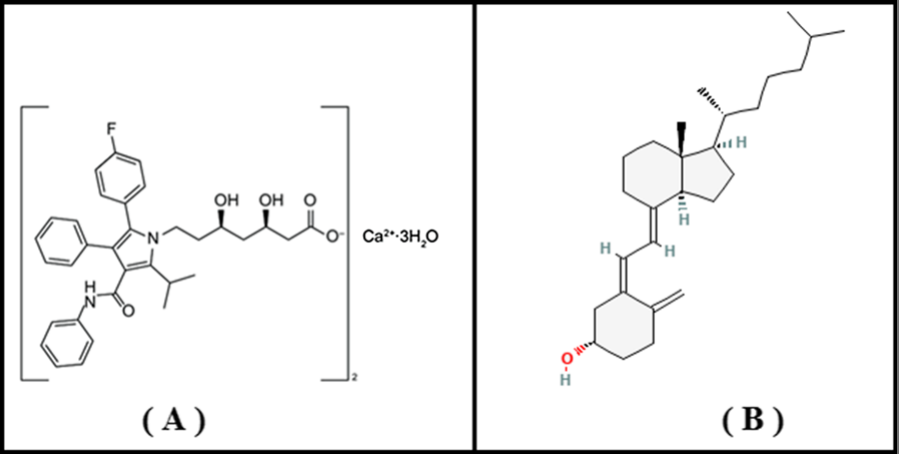
Chemical structure of atorvastatin calcium (**A**) and vitamin D_3_ (**B**)

Atorvastatin calcium (Fig. 1a) is an important member of statins group which is the first line treatment of hyperlipidemia because of their effectiveness. Statins inhibit hydroxyl-methyl glutaryl Co-A reductase, a rate-controlling enzyme in cholesterol biosynthesis, reducing the cholesterol production which positively affects the rates of cardiovascular complications and general mortality in patients with coronary artery disease [1].

The vitamin D is a group of fat soluble vitamins which are vital for nearly all human body systems like immune, myocardial systems, pancreatic beta cells, neurons and so its participation in many of metabolic disorders [2]. In humans, the most important compounds in this group are vitamin D_3_ (cholecalciferol) and vitamin D_2_ (ergocalciferol). vitamin D_3_ or cholecalciferol (Fig. 1b) coordinates calcium regulation in the body [3] and affects impressively muscle duties and overall health especially with long use [4]. It’s very important to determine and quantify vitamin D_3_ levels in many cases which suffer from muscle dysfunctions [5].

A clinical trial was performed to study the role of statins on muscle work, it was found that 9.4% of the patients taking 80 mg/day atorvastatin over 6 months was developing muscle pain in comparison with 4.6% of placebo patients [6]. Statin-associated muscle symptoms (SAMS) are considered as critical adverse effects of prolonged statin therapy such as myopathy, myalgia, and rhabdomyolysis. Vitamin D has been linked with muscle health and performance. Myalgia can be controlled by adjustment of serum vitamin D level [7].

Atorvastatin calcium has been analyzed by many techniques such as HPLC [8–14] and spectrophotometrically [15–19]. While, vitamin D_3_ was determined by HPLC with UV detection [20–24] or mass spectrophotometry [25–28] and spectrophotometrically using iodine complex [29]. Atorvastatin calcium and vitamin D_3_ have been determined together using reversed phase-HPLC with UV detection at 265 nm using mobile phases of harmful and toxic solvents of methanol:acetonitrile (50:50) [30] and at 252 nm using mobile phase of acetonitrile:methanol ratio of 75:25 v/v, pH of 3.5 is adjusted with orthophosphoric acid [31] thus generating toxic residues and waste. Green analytical chemistry (GAC) was introduced in year 2000 to reduce or to remove the harmful effects of analytical practices. It is a challenge to increase the quality of results and improve environmental friendliness of analytical procedure. For the evaluation of the analytical greenness, we have applied Green Analytical Procedure Index (GAPI) [32] which is a new tool used for assessment of the greenness of analytical procedures. To classify the greenness of each stage of an analytical procedure, the GAPI tool applies a pictogram using a color scale from green through yellow to red depicting low, medium to high impact, respectively. GAPI estimation system fundamentally covers three categories; sampling, solvents and equipments which are subdivided into 15 parts of assessment [33].

Also, the Analytical GREEnness Metric Approach (AGREE) assessment tool has been reported recently [34] for evaluation of greenness degree of the proposed method according to the 12 main principles of green analytical chemistry in form of score from zero to one.

Here, we have developed a green and eco-friendly HPLC method for analysis of atorvastatin calcium and vitamin D_3_ either in pure form or in pharmaceutical formulation.

## Experimental

### Chemicals and reagents

Atorvastatin calcium working standard (99.4% purity) was supplied and certified by EIPICO, Egypt and its commercial product (Lipitor tablets 10 mg, Pfizer Company for pharmaceuticals, Egypt) was purchased from Egyptian market. Vitamin D_3_ standard (> 98% purity) was purchased from Sigma-Aldrich (Germany) and its commercial product (Breva tablets 10,000 I.U) produced by Vortex pharma company (Egypt). Ethanol (HPLC grade) and methanol (Analytical grade) were provided by Darmstadt, Merck (Germany). Phosphoric acid (Analytical grade) was purchased from Sigma Aldrich (Germany).

### Instrumentation and chromatographic conditions

HPLC analyses were performed on Alliance 2695 HPLC system which composed of a quaternary gradient pump, an auto sampler, a column oven, and a photodiode array detector 2996 (Waters, USA). The separation was performed on symmetry column C_18_ (100–4.6 mm, 3.5 µm) (Waters, Ireland) using a mixture consisting of 0.1% ortho-phosphoric acid (OPA) pH = 2.16 and ethanol as the mobile phase in gradient manner. The mobile phase was pumped at a flow rate of 1 ml/min while the column temperature was maintained at 40 °C. Detection was monitored at wavelengths of 246 and 264 nm for atorvastatin calcium and vitamin D_3_ respectively. Injection volume was set as 20 µl.

### Preparation of stock solutions

In a 100 ml volumetric flask, stock solutions of atorvastatin calcium, vitamin D_3_ (0.1 mg/ml) were prepared by dissolving 10 mg of each drug separately in methanol, sonicate for 10 min, and then the volumes were completed with methanol.

Working standard solutions were made by diluting aliquots of the stock solution with methanol to get concentrations of 1, 5, 10, 20, 30 and 40 μg/ml for atorvastatin calcium and 1, 2, 4, 6 and 8 μg/ml for vitamin D_3_.

### Construction of calibration curves

The calibration charts were set as a relation between the peak areas and the corresponding injected concentrations of atorvastatin calcium and vitamin D_3_.

### Application to pharmaceutical dosage form

For atorvastatin calcium and vitamin D_3_, 10 tablets of Lipitor^®^ (10 mg) or Breva^®^ (10,000 IU) respectively were powdered. weights of the powders equivalent to 0.15 gm atorvastatin calcium and 0.6 g vitamin D_3_ were transferred into 50 ml volumetric flasks and then dissolved in methanol. The flasks were left for 15 min in the sonicator, then filtered into dry conical flasks, completed to 50 ml with methanol and then 20 µl injected.

### Method validation

The validation of this analytical method was carried in accordance with the International Conference on Harmonization (ICH) instructions that include Linearity, precision, specificity, accuracy, robustness, limit of detection and limit of quantitation [35].

## Results and discussion

We have succeeded here in reaching an acceptable compromise between increasing the quality of results and improving environmental friendliness of analytical methods.

The two drugs have been separated in less than 10 min using a green mobile phase of 0.1% ortho-phosphoric acid (OPA) (pH = 2.16) and ethanol in gradient manner.

Different greenness assessment tools as GAPI and AGREE were used to assess the greenness degree of our developed method.

### Optimization of HPLC conditions

**Fig. 2:**
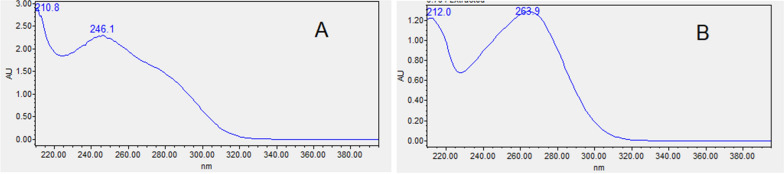
Absorption Spectra of atorvastatin calcium (**A**) and vitamin D_3_ (**B**)

Different conditions were studied and optimized to increase the resolution and sensitivity of the proposed HPLC method for the separation of atorvastatin calcium and vitamin D_3_. Detection was selected at wavelengths of 246 and 264 nm for atorvastatin calcium and vitamin D_3_ respectively because they achieved the maximum absorption for both (Fig. 2A and B).

**Fig. 3:**
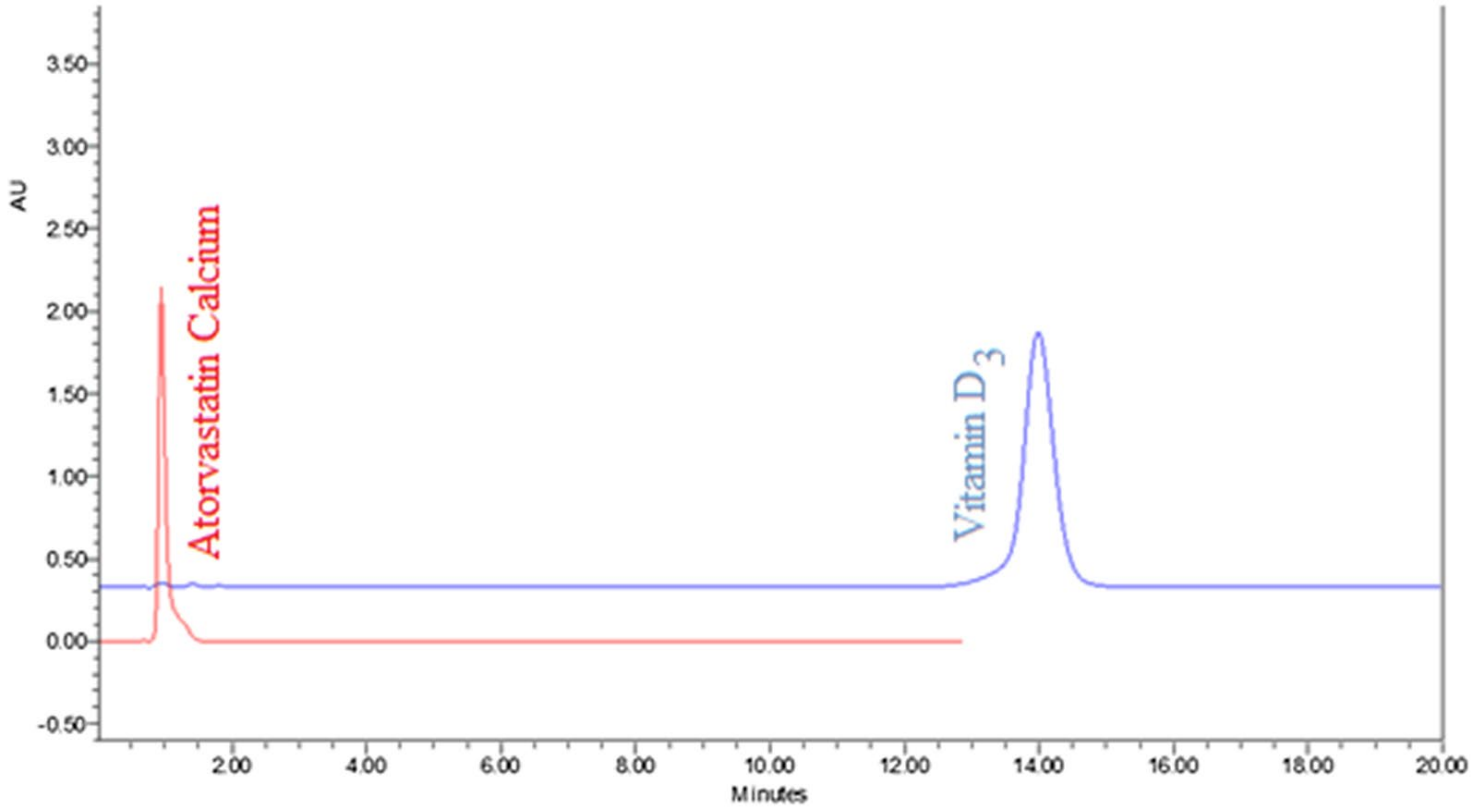
Chromatogram of 20 µl injection of standard solutions of atorvastatin calcium (0.1 mg/ml) and vitamin D_3_ (0.1 mg/ml) using 0.1% orthophosphoric acid and ethanol as the mobile phase [50%:50%] at a flow rate 1 ml/min with column temperature was maintained at 40 °C and detection at 246 and 264 nm for atorvastatin calcium and vitamin D_3_ respectively

A green mobile phase (aquatic acidic modifier: Ethanol) was used for separation in addition to two essential parameters;

1. pH of separation media:
  According to Henderson–Hasselbalch equation (pH = pKa + Log [A^−^]/[HA]); pH of the system is important factor that's calculated by values of pKa of species in our solution for adjusting pH of mobile phase to be an acidic media using non-Polar column to separate the peak of “atorvastatin calcium” making it in ionized form. The optimum pH was approximately 2.16.
2. Elution strength of mobile phase (Gradient elution):
  As, vitamin D_3_ is highly hydrophobic so, it could be separated by changing ratios of mobile phase (extremely increase in “ethanol” portion).
  We have tried different mixtures of 0.1% ortho-phosphoric acid (OPA) (pH = 2.16) or 0.1% v/v formic acid (PH = 2.7) and ethanol in an isocratic or gradient elution. In case of isocratic elution, atorvastatin was early eluted in contrary to the highly retained vitamin D_3_ (Fig. 3). Columns used in trials included symmetry column (4.6 × 100 mm, 3.5 µm) (Waters, Ireland), XTerra column C18 (4.6 × 100 mm, 5 µm (Waters, USA).

**Fig. 4:**
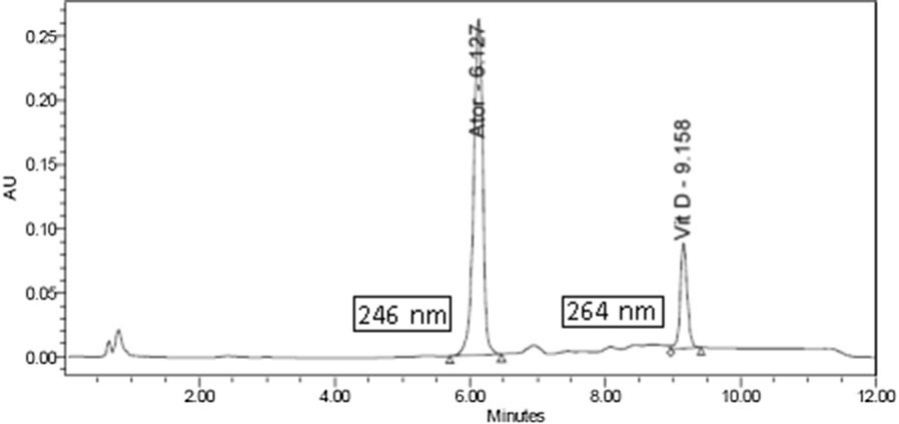
Chromatogram of 20 µl injection of standard solutions of atorvastatin calcium (0.1 mg/ml) and vitamin D_3_ (0.1 mg/ml) using 0.1% orthophosphoric acid and ethanol as the mobile phase in gradient matter at a flow rate 1 ml/min with column temperature was maintained at 40 °C and detection at 246 and 264 nm for atorvastatin calcium and vitamin D_3_ respectively

The best chromatographic separation was achieved using symmetry column (4.6 × 100 mm, 3.5 µm) and ethanol with 0.1% ortho phosphoric acid in gradient manner (Fig. 4, Table 1). So, gradient elution was selected for simultaneous determination of both drugs in a reasonable time with good peak symmetry and high resolution.

**Table 1:** Gradient mode of the mobile phase

Time (min)	0.1% OPA	Ethanol
0	60	40
3	60	40
7	5	95
10	5	95
10.1	60	40
12	60	40

### Method validation

#### Linearity

Good linearity was achieved between the peak areas of atorvastatin calcium, vitamin D_3_ and the corresponding concentration ranges which was confirmed by the high correlation coefficient as mentioned in Table 2.

**Table 2:** Regression and validation data for the determination of atorvastatin calcium and vitamin D_3_

Parameters	Proposed method	
	Atorvastatin calcium	Vitamin D_3_
Wavelength (nm)	246	264
Linearity range (µg/ml)	5–40	1–8
LOD (µg/ml)	0.48	0.04
LOQ (µg/ml)	1.43	0.12
Regression equation (y = ax + b)		
Slope (a)	11,949	13,724
Intercept (b)	60,912	− 1251.5
Coefficient of determination (r^2^)	0.9998	0.9995
Robustness (% R ± % RSD)		
▲λ (± 2 nm)	99.99 ± 0.27	99.49 ± 0.45
Ethanol (± 1%)	98.42 ± 1.75	99.13 ± 0.77
PH (± 0.1)	97.13 ± 2.57	99.34 ± 0.63

#### Limit of detection and limit of quantitation

LOD and LOQ values were calculated according to the following equations, and the obtained results were shown in Table 2.

$$ {\text{LOD}} = {3}.{3}\sigma /{\text{S}} $$

$$ {\text{LOQ}} = {1}0\sigma /{\text{S}} $$

where (σ) is the standard deviation of the intercept of the regression line and (S) is the slope of the calibration curve.

#### Specificity and accuracy

**Fig. 5:**
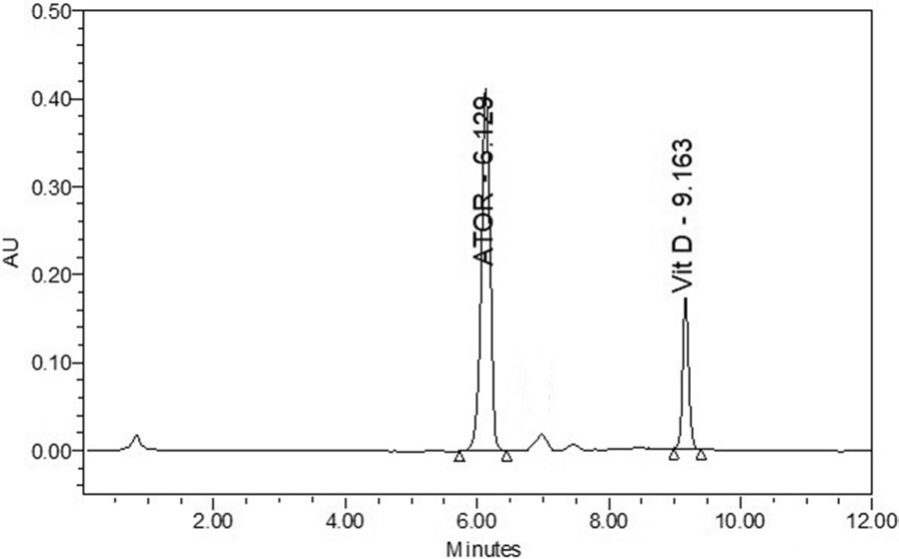
Chromatogram showing separation of atorvastatin calcium and vitamin D_3_ in their tablets utilizing the optimized HPLC method of study

Specificity is defined as how the proposed method can give the same drug response in presence of tablet excipients. A placebo was prepared containing excipients which could be present in the tablet formulation to prove accuracy. The obtained results, as in Table 3, showed that the method of study was highly selective to analyze atorvastatin calcium and vitamin D_3_ in their tablets without any effect or interference from the excipients (Fig. 5).

**Table 3:** Accuracy results of the proposed HPLC–UV method

Drug	Pure added (µg/ml)	Pure found (µg/ml)	Pure recovery* (R%)
Atorvastatin calcium	10	9.82	98.19
	20	19.87	99.37
	40	40.33	100.84
Vitamin D_3_	2	1.96	97.97
	4	4.06	101.46
	8	8.04	100.46

^*^Mean of three determinations

Also, the obtained results were compared with results of the reported method [30]. It was found that the calculated t and F values were less than the tabulated ones so there is no significant difference between the proposed method and the reported one as summarized in Table 4.

**Table 4:** Determination of atorvastatin calcium and vitamin D_3_ by the proposed method in comparison with the reported method [30]

Parameter	Proposed method		Reported method [30]	
	Atorvastatin calcium	Vitamin D_3_	Atorvastatin calcium	Vitamin D_3_
Mean recovery^$^ % ± SD	102.87 ± 0.97	101.35 ± 2.24	100.5 ± 0.04	101.7 ± 0.15
RSD (%)	0.95	2.21	0.04	0.15
Student-t-test*	0.072		0.878	
F-test*	0.003		0.009	
N	5		3	

^$^Mean of five determinations for proposed method and three determinations for reported method

^*^Tabulated values of t and F values at p = 0.05 are 2.776 and 19.2

### Precision

An acceptable precision of the method was proved through an intra-day and inter-day precision confirming low value of RSD% for atorvastatin calcium and vitamin D_3_ (< 2%) as in Table 5.

**Table 5:** Precision results of the proposed HPLC–UV method

Drug	Added concentration (µg/ml)	Intra-day precision		Inter-day precision	
		Mean*% ± SD	RSD%	Mean*% ± SD	RSD%
Atorvastatin calcium	10	98.44 ± 0.27	0.28	98.79 ± 0.47	0.48
	20	101.26 ± 0.89	0.88	102.06 ± 1.04	1.02
	40	99.49 ± 0.31	0.32	100.14 ± 0.74	0.74
Vitamin D_3_	2	98.41 ± 0.49	0.50	98.77 ± 0.59	0.60
	4	101.43 ± 0.45	0.44	101.91 ± 0.62	0.60
	8	100.21 ± 0.32	0.32	100.51 ± 0.39	0.39

*Mean of three determinations

#### Robustness

Robustness is defined as the ability of the method to remain unchanged with small but deliberate changes in the experimental conditions such as wavelength, organic mobile phase and pH. Small changes in such conditions didn’t have any obvious effects on the optimum results produced by the proposed method as shown in Table 2.

### Greenness assessment tools

Using different greenness assessment tools as GAPI and AGREE tools, our developed method has the highest greenness degree with increasing quality of results over the other reported HPLC methods [30, 31] as summarized in Table 6.

**Table 6:** Comparison of the proposed analytical method to the reported HPLC methods

	Proposed green HPLC method	Reported method [30]	Reported method [31]
Technique	Green HPLC–UV	RP-HPLC–UV	RP-HPLC–UV
Linearity range (µg/ml) Atorvastatin calcium	5–40	50–500	8–28
Vitamin D_3_	1–8	0.13–1.25	2–7
Organic Solvent	0.1% ortho-phosphoric acid (OPA) (pH = 2.16), ethanol	Methanol, Acetonitrile (50%:50%) pH adjusted to 4 with 0.1% formic acid	Acetonitrile, Methanol (75%: 25%) pH adjusted to 3.5 adjusted with phosphoric acid
Run time (min)	12	15	20
Column	Symmetry column C_18_ (100 × 4.6 mm, 3.5 µm)	Enable C_8_-column (15 cm × 4.6 mm, 5 μm)	Phenomenex, Luna C_18_ (250 × 4.6 mm, 5 μm)
GAPI assessment			
AGREE assessment			

In GAPI, the sample preparation divided into; collection was on-line when the sample doesn’t need more preservation or transportation and the storage was under normal conditions in presence of green solvents like ethanol. For reagents and solvents; the amount of solvents used was 10–100 ml per run. The produced health hazards was little (score 0–1) and safety hazards of score 2–3. For instrumentation, the consumed energy was < 1.5 kWh per sample with no occupational hazards and little waste.

For AGREE, online sampling procedure was performed by HPLC system with score of 0.48. Minimal sample size was achieved (0.1 mg/ml) with score of 1. In-situ sample preparation was measured with score equals 0.66. Besides that, preparation of the sample was in less than 3 steps such as sonication and dilution leading to score of 1. Our developed method is semi-automatic, miniaturized method with score equals to 0.75.

There is no need to use derivatizing agent or toxic reagents (score = 1) and no threats with maximum safety of operator (score = 1). Moreover; waste volume was 12 ml/run (score = 0.36). Energy consumption was little (less than 1.5 kWh per sample (score = 0.5)). The proposed method was capable to determine 10 analytes per hour (score = 0.51) and can use bio-based reagents from renewable sources such as ethanol (score = 0.5).

## Conclusion

The developed method provides eco-friendly approach for analysis of atorvastatin calcium and vitamin D_3_ that is dependant on usage of extremely green solvents such as ethanol and water. The proposed method was successfully applied for determining the investigated drugs in their commercial dosage forms. GAPI and AGREE assessment tools were used for evaluation of greenness degree confirming that the proposed method was green with high economic impact. The method was validated according to ICH recommendations. The studied validation parameters were within their acceptable ranges giving more sensitivity and applicability to the method.

## Data Availability

The datasets used and/or analysed during the current study are available from the corresponding author on reasonable request.
